# Casein Kinase 2 Dependent Phosphorylation of Neprilysin Regulates Receptor Tyrosine Kinase Signaling to Akt

**DOI:** 10.1371/journal.pone.0013134

**Published:** 2010-10-01

**Authors:** Martin Siepmann, Sathish Kumar, Günter Mayer, Jochen Walter

**Affiliations:** 1 Department of Neurology, Molecular Cell Biology, University of Bonn, Bonn, Germany; 2 Limes-Institute, Chemical Biology c/o Kekulé-Institute for Organic Chemistry and Biochemistry, University of Bonn, Bonn, Germany; University of Birmingham, United Kingdom

## Abstract

Neprilysin (NEP) is a type II membrane metalloproteinase that cleaves physiologically active peptides at the cell surface thus regulating the local concentration of these peptides available for receptor binding and signal transduction. In addition, the cytoplasmic N-terminal domain of NEP interacts with the phosphatase and tensin homologue deleted on chromosome 10 (PTEN) thereby regulating intracellular signaling via Akt. Thus, NEP serves dual functions in extracellular and intracellular signal transduction. Here, we show that NEP undergoes phosphorylation at serine residue 6 within the N-terminal cytoplasmic domain. *In vitro* and cell culture experiments demonstrate that Ser 6 is efficiently phosphorylated by protein kinase CK2. The phosphorylation of the cytoplasmic domain of NEP inhibits its interaction with PTEN. Interestingly, expression of a pseudophosphorylated NEP variant (Ser6Asp) abrogates the inhibitory effect of NEP on insulin/insulin-like growth factor-1 (IGF-1) stimulated activation of Akt. Thus, our data demonstrate a regulatory role of CK2 in the interaction of NEP with PTEN and insulin/IGF-1 signaling.

## Introduction

Neutral endopeptidase 24.11 (NEP, neprilysin, CD10) is a zinc metallopeptidase that has broad substrate specificity and cleaves several important peptides, including bombesin, ET-1, and the Alzheimer-associated amyloid β-peptide, thereby regulating their turnover and physiological and pathophysiological activities [1], [2], [3], [4]. NEP is a type II integral membrane protein with a small N-terminal cytoplasmic tail, a single transmembrane domain and a larger extracellular C-terminal domain that contains the catalytic center [1]. NEP is post-translationally modified by N-glycosylation of the luminal/extracellular domain that regulates its subcellular transport and enzymatic activity [5], [6].

Accumulating evidence indicates that NEP is also involved in tumor formation. The expression of NEP is downregulated in androgen-independent prostate cancer cells [7], [8], [9], [10]. In addition, overexpression of NEP in tumor cells could inhibit proliferation [9].

Beside the functions of NEP in the metabolism of extracellular peptides, recent studies also indicate a role of its cytoplasmic domain in intracellular signaling [10]. The cytoplasmic N-terminal domain of NEP interacts with the scaffolding proteins Ezrin/Radixin/Moesin (ERM) [7], [11] that link particular plasma membrane proteins to the actin cytoskeleton at specialized domains of plasma membranes such as microvilli and cell–cell or cell–substrate adhesion sites [12], [13].

In addition, the cytoplasmic domain of NEP could also interact with PTEN (Phosphatase and tension homologue deleted on chromosome 10), a major tumor suppressor protein [14], [15]. PTEN has phosphatase activity and dephosphorylates phosphatidylinositol (3,4,5)-trisphosphate (PIP_3_) to phosphatidylinositol (4,5)-bisphosphate (PIP_2_) in cellular membranes. Thus, PTEN antagonizes the activity of phosphatidylinositide-3-kinase (PI3K) that is activated by several receptor tyrosine kinases (RTKs) upon binding of ligands, like insulin, insulin-like growth factor-1 and epidermal growth factor [16], [17], [18]. PIP_3_ dependent signaling involves recruitment of proteins that contain pleckstrin homology (PH) and PH-like domains to the plasma membrane [19]. One important member of PIP_3_ binding proteins is the protein kinase (PK) B/Akt that phosphorylates several protein substrates and thereby regulates a variety of cellular functions, including cell survival, differentiation and proliferation [20], [21]. Akt could exert anti-apoptotic activity by phosphorylation and inhibition of pro-apoptotic proteins [22], [23]. Accordingly, enhanced expression of PTEN can induce apoptosis by inhibition of the Akt pathway [24], whereas decreased expression or inactivation of PTEN in tumors results in increased activity of Akt [25], [26].

The catalytic center of PTEN is localized within its N-terminal domain, whereas the C-terminal C2 domain mediates binding to phospholipids and recruitment to cellular membranes [27], [28]. The C-terminal tail of PTEN can be phosphorylated on serine and threonine residues by protein kinase CK2 which negatively regulates its phosphatase activity [29], [30]. The activity and/or expression of CK2 is increased in several cancers [31], [32], [33], [34], and transgenic overexpression of CK2 results in formation of tumors and lymphomas [33], [35]. Interestingly, CK2 also phosphorylates NEP *in vitro* [36]. However, the site and a functional role of phosphorylation in the regulation of NEP remained unclear.

Here, we sought to characterize the phosphorylation of NEP and its implications in insulin-dependent signal transduction. We demonstrate that NEP is efficiently phosphorylated by CK2 on serine residue 6 within its cytoplasmic N-terminal domain *in vitro* and in cultured cells. Phosphorylation strongly inhibits the interaction of NEP with PTEN and impairs the inactivation of Akt upon stimulation of RTKs with insulin or insulin-like growth factor-1. These data provide novel insight into molecular mechanisms that regulate the NEP dependent activity of PTEN and support important roles of CK2 and NEP in insulin/IGF-1 dependent signaling via Akt.

## Materials and Methods

### Antibodies, peptides, and chemicals

The following primary antibodies were used: mouse monoclonal NEP (F-4; western immunoblotting [WB] 1∶1,000), mouse monoclonal CK2α (1AD9; immunofluorescence [IF] 1∶200), rabbit polyclonal calnexin (H-70; WB 1∶1,000; IF 1∶300) (all Santa Cruz Biotechnology, Santa Cruz, CA, USA), mouse monoclonal c-myc (9E10; WB 1∶1,000; IF 1∶500; immunoprecipitation [IP] 1∶200; Abcam, Cambridge, UK), rabbit polyclonal phospho-Akt (serine 473; #9271; WB 1∶1,000; Cell Signaling Technology, Danvers, MA, USA), rabbit monoclonal Akt (AW24; WB 1∶3,000; Millipore, Billerica, MA, USA), mouse monoclonal GFP (clones 7.1 and 13.1; WB 1∶3,000; IF 1∶1,000; Roche, Mannheim, Germany), rabbit polyclonal TGN46 (T7576; IF 1∶300; Sigma-Aldrich). HRP coupled streptavidin protein (ab7403; WB 1∶1,000) was obtained form Abcam, Cambridge, UK. Secondary HRP-conjugated antibodies were purchased form Sigma-Aldrich and used in a 1∶50,000 dilution. Secondary Alexa 594- and Alexa 488-conjugated antibodies were purchased form Invitrogen and used in a 1∶500 dilution. Peptides representing the N-terminal domain (aa 2–23) of NEP and conjugated with biotin on a C-terminal lysine were obtained from Peptide Specialty Labs (PSL, Heidelberg, Germany). Radiochemicals were purchased from Hartmann Analytic. 2-Dimethylamino-4,5,6,7-tetrabromo-1H-benzimidazole (DMAT), 4′,6-Diamidino-2-phenylindole dihydrochloride (DAPI), insulin-like growth factor-1 (IGF-1) and insulin were from Sigma-Aldrich.

### cDNAs and fusion proteins

The cDNAs encoding human NEP and Ezrin were kindly provided by Drs. Takaomi Saido and Monique Arpin. The cDNA of human PTEN was obtained from the German Resource Center for Genome research (RZPD, Berlin, Germany). A C-terminal myc/his-tagged variant of NEP was generated by PCR using the oligonucleotides 5′-tttggtaccatgggcaagtcagaaagtc-3′ and 5′-cccgcggccgcccaaacccggcac-3′. The resulting fragment was subcloned into the KpnI/NotI restriction sites of pcDNA4 Myc/His containing a Zeocin resistance gene (Invitrogen, Karlsruhe, Germany). The phosphorylation site mutants of NEP were generated by PCR using the following forward oligonucleotides: S6A: 5′-tttggtaccatgggcaagtcagaagctcagatg-3′; T15A: 5′-tttggtaccatgggcaagtcagaaagtcagatggatataactgatatcaacgctccaaag-3′. The oligonucleotide 5′-cccgcggccgcccaaacccggcac-3′ was used as reward primer for all constructs. The resulting PCR fragments were subcloned into the KpnI/NotI restriction sites of the expression vector pcDNA4 Myc/His Zeo.

NEP variants tagged with green fluorescent protein (GFP) were generated by PCR using the following primers: 5′-tttggtaccatgggcaagtcagaaagtc-3′, 5′-cccggatccaatgcatagagtgcgatc-3′. The resulting fragments were subcloned into the KpnI/BamHI restriction sites of pEGFP-N1 (Clontech, Mountain View, CA, USA). The serine 6 to aspartate mutant was generated by PCR using oligonucleotides 5′-tttggtaccatgggcaagtcagaagatcagatg-3′ and 5′- cccggatccaatgcatagagtgcgatc-3′.

Fusion proteins of the NEP N-terminus (aa 1-28) and glutathione S-transferase (GST) were generated by PCR using the primers 5′-ccgaattcatgggcaagtcag-3′ and 5′-cccgtcgacctactccagtggagtcc-3′. The resulting fragments were cloned into the EcoRI/SalI restriction sites of pGEX-5X-1 (GE Healthcare, Little Chalfont, UK). A fusion protein of the maltose binding protein (MBP) and the N-terminus of Ezrin (amino acids 1–310; MBP-Ezrin-NT) was generated using primers 5′-cccgaattcatgccgaaaccaatcaat-3′ and 5′-tttgtcgacttaccgggcctgggccttcat-3′. The resulting fragment was cloned into the EcoRI/SalI restriction sites of pMAL-c2 (Promega, Mannheim, Germany).

The fusion protein of GST and the C-terminal domain (aa 367–403) of PTEN (GST PTEN-CT) was generated by PCR using primers 5′-cccgaattcgatgttagtgacaatg-3′ and 5′-cccctcgagatcagacttttgtaatttgtg-3′. The resulting fragment was cloned into the EcoRI/XhoI restriction sites of pGEX-5X-1 (GE Healthcare, Little Chalfont, UK). All fusion proteins were expressed in *Escherichia coli* DH5α. GST and MBP fusion proteins were precipitated by GSH-resin and Amylose-resin (GE Healthcare, Little Chalfont, UK) respectively according to the supplier's instructions. All constructs were verified by sequencing of both strands.

### Cell culture, transfection, and treatment

Human embryonic kidney (HEK) 293 and human HeLa cells (both ATCC, Manassas, VA, USA) were cultured in Dulbecco's modified Eagle's medium (DMEM) supplemented with 10% fetal calf serum at 37°C in a 5% CO_2_ atmosphere. DMAT was dissolved in DMSO at concentrations of 10 mM and applied to cells at a final concentration of 20 µM. Control cells were incubated with the carrier alone. Transfections of the cells with cDNA constructs were carried out using Lipofectamine 2000 (Invitrogen, Karlsruhe, Germany) according to supplier's instructions. Cells were analyzed 36–48 hrs after transfection. For the activation of Akt, HEK293 cells were starved in DMEM without serum for 16 hrs and then treated with 100 nM insulin or 1 ng/ml (0.13 nM) IGF-1 (Sigma-Aldrich, Munich, Germany) for 20 min.

### Cell lysis, immunoprecipitation and Western immunoblotting

Cells were washed twice with PBS, collected by scraping from the culture dish and lysed for 30 min on ice (lysis buffer: 50 mM Tris/HCl, pH 7.6, 150 mM NaCl, 2 mM EDTA, 1% Triton X-100, 1% Igepal, Complete protease inhibitor mixture, PhosSTOP phosphatase inhibitor (Roche, Mannheim, Germany)). The lysate was then cleared by centrifugation (16,000× g, 4°C, 30 min). Proteins were immunoprecipitated from cleared lysates using primary antibodies (5 µg/ml) and protein G-conjugated sepharose (GE Healthcare, Little Chalfont, UK) for 3 hrs at 4°C. Precipitates were washed three times with washing buffer (50 mM Tris/HCl, pH 7.4, 500 mM NaCl, 2 mM EDTA, 0.2% Igepal) for 10 min at 4°C. Precipitates were collected by centrifugation (4,000× g, 4°C, 5 min) and eluted by incubation with SDS sample buffer (25 mM Tris/HCl, pH 6,8, 10% Glycerin, 1,5% SDS, 20 mM DTT) for 10 min at 95°C.

Proteins were then separated by SDS-PAGE, electro-transferred to nitrocellulose membranes (Whatman, Dassel, Germany), and detected with appropriate antibodies by enhanced chemiluminescence (ECL)-imaging (ChemiDoc XRS, Bio-Rad, Munich, Germany).

### *In vivo* and *in vitro* phosphorylation of NEP

Phosphorylation of NEP in cultured cells was carried out as described earlier [37]. Briefly, cells were grown on 28 cm^2^ dishes to subconfluency and then incubated for 1 h in phosphate-free media (Sigma-Aldrich, Munich, Germany). The media was aspirated, and phosphate-free media was added, together with 0.25 mCi of [^32^P] orthophosphate (Hartmann Analytic, Braunschweig, Germany) for 2 hrs at 37°C. The conditioned media were aspirated, cells washed three times with PBS and immediately lysed on ice with lysis buffer. Cell lysates were centrifuged for 10 min at 16,000× *g*, and supernatants were immunoprecipitated with anti-myc antibodies. Radiolabeled proteins were separated by SDS-PAGE as described above and detected by autoradiography and western immunoblotting. *In vitro* phosphorylation assays with recombinant CK2 (alpha and beta subunit; New England Biolabs) were carried out according to the manufacturer's instructions. Phosphorylation reactions were started by addition of 10 µM [γ-^32^P] ATP and allowed to proceed for 10 min at 32°C. Reactions were stopped by the addition of 5 mM EDTA. GST-fusion proteins were precipitated by GSH-sepharose (GE Healthcare, Little Chalfont, UK) and separated by SDS-PAGE.

### Immunocytochemistry and fluorescence microscopy

Cells were grown on poly-L-lysine-coated glass coverslips to 50–80% confluency and fixed with 4% paraformaldehyde/PBS at room temperature and processed for immunofluorescence as described previously [38]. Bound primary antibodies were detected by Alexa 488- or Alexa 594-conjugated secondary antibodies (Invitrogen, Karlsruhe, Germany). GFP-tagged proteins were detected directly by fluorescence microscopy. The samples were analyzed with an Axiovert 200 Fluorescence Microscope (Carl Zeiss, Jena, Germany).

### Surface plasmon resonance measurements

Surface plasmon resonance (SPR) measurements were carried out on a Biacore3000 (GE Healthcare, Little Chalfont, UK) as described previously [39], [40]. Synthetic peptides with a C-terminal biotin-label that represent amino acids 2–23 of the human NEP cytosolic domain with serine 6 in phosphorylated or non-phosphorylated state, were coupled to streptavidine (SA) chip (GE Healthcare, Little Chalfont, UK) until ∼1000 response units (RU) were reached. The chip was washed with HEPES buffer (10 mM, pH 7.4) containing 150 mM NaCl and 0.005% Surfactant P20 (HBS-P buffer; GE Healthcare, Little Chalfont, UK) at 25°C and a constant flow rate of 30 µl/min. To avoid bulk effects, a reference surface (no coupled peptide) was continuously subtracted from the signal of the active surface. To calculate the association and dissociation rates of the respective sensograms and the resulting equilibrium dissociation constant (K_D_) the BIAevaluation 3.1 software was used [41], [42].

### Pull-down assays with immobilized peptides

The above described biotin-labeled NEP NT peptides were coupled to Streptavidin (SA)-agarose beads (GE Healthcare, Little Chalfont, UK) and incubated with GST PTEN-CT (2 µM), MBP Ezrin-NT (100 nM) or CK2 (30 nM; alpha and beta subunit; New England Biolabs, Frankfurt, Germany) in binding buffer (10 mM Tris/HCl, pH 7.4, 150 mM NaCl, 0.4% Igepal) for 2 hrs. After washing with the same buffer, bound proteins were eluted with SDS sample buffer, separated by SDS-PAGE, and detected by Western immunoblotting with polyclonal antibodies against MBP, (MBP Ezrin-NT), PTEN (GST PTEN-CT) or monoclonal antibody against CK2α.

### Biotin-labeling of cell surface proteins

Cells were washed three times with ice-cold PBS and incubated on ice with PBS containing 0.5 mg/ml EZ-Link sulfo-*N*-hydroxysuccinimide-biotin (Thermo, Bonn, Germany) for 30 min. Cells were then washed three times with ice-cold PBS supplemented with 20 mM glycine and finally lysed on ice with lysis buffer. Biotinylated proteins were precipitated from cleared lysates with SA-agarose beads (GE Healthcare, Little Chalfont, UK) and separated by SDS-PAGE. The respective proteins were then detected by Western immunoblotting.

### Data Analysis and Statistics

For enhanced chemiluminescence detection, signals were measured and analyzed using an ECL imager (ChemiDoc XRS; Bio-Rad, Munich, Germany) and the Quantity One software package (Bio-Rad, Munich, Germany). Statistical analysis was carried out using Student's *t* test. Significance value: ^*^, *p*<0.05.

## Results

### CK2 phosphorylates NEP within its cytoplasmic domain at serine 6

**Figure 1:**
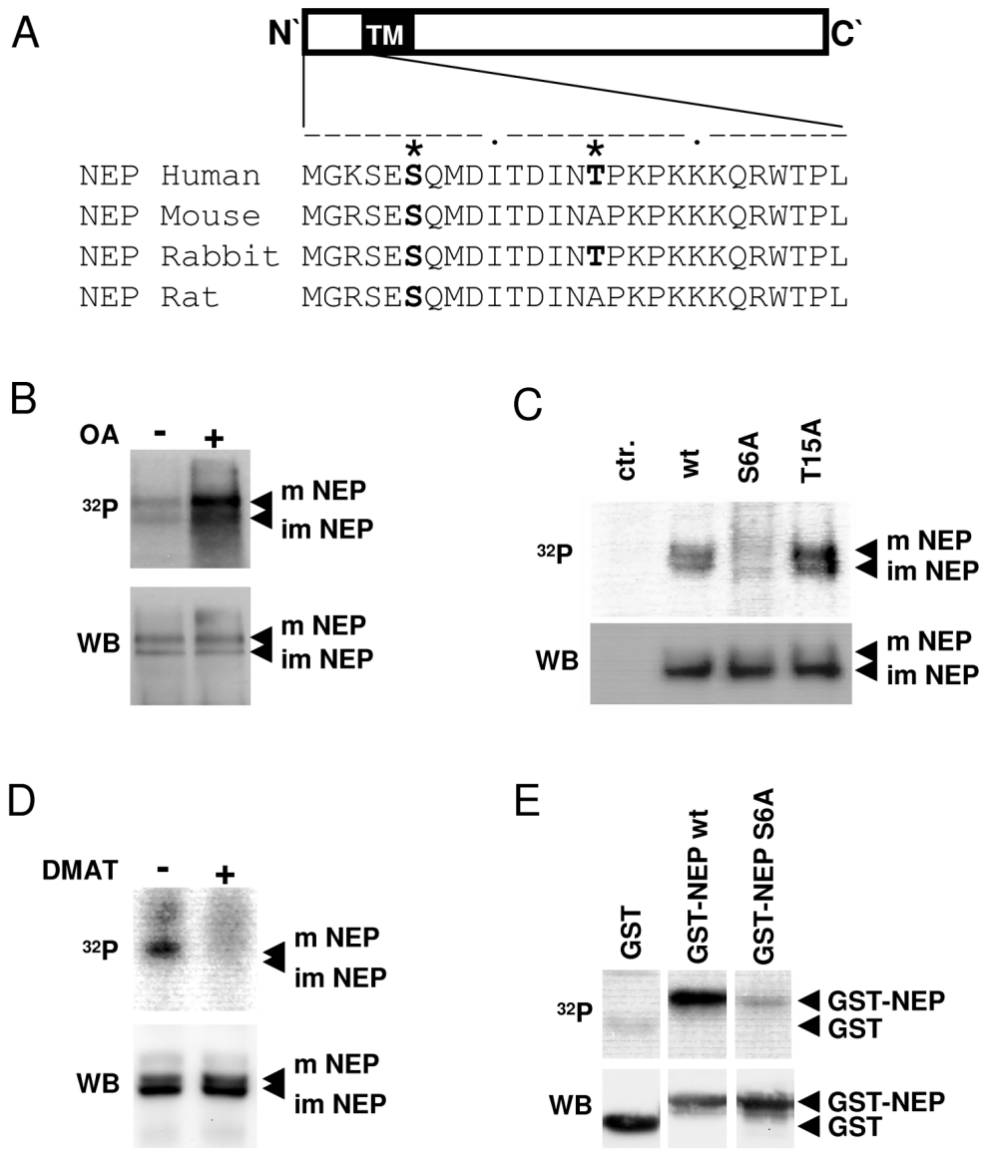
NEP is phosphorylated by CK2 at serine 6. *A*, Alignment of the N-terminal amino acid sequences of NEP from several mammalian species. Potential phosphorylation sites predicted by the NetPhos 2.0 server (with score >0.9) are indicated by asterisks. *B*, *C*, *D* HEK293 cells transiently expressing myc-tagged NEP wt or respective mutants were labeled with [^32^P] orthophosphate in presence or absence of okadaic acid (OA) (*B*) or DMAT (*D*) as indicated. After immunoprecipitation, the radiolabeled proteins were detected by autoradiography (upper panels). Subsequently, NEP was detected by immunoblotting with antibody 9E10 on the same membrane (lower panel). *E*, GST-fusion proteins representing amino acid 1–28 of the NEP N-terminus (GST-NEP) were incubated with CK2 in the presence of [γ-^32^P]ATP. The radiolabeled proteins were detected by autoradiography (upper panels). Subsequently, GST-fusion proteins were detected by immunoblotting with antibody against GST on the same membrane (lower panel). m = mature; im = immature; ctr. = untransfected cells.

The N-terminal cytoplasmic domain of NEP contains several potential phosphorylation sites (Fig. 1A). To assess whether NEP undergoes phosphorylation, HEK293 cells transiently expressing myc-tagged variants of full-length NEP were labeled with ^32^P-orthophosphate. Phosphate incorporation into non-glycosylated immature (∼90 kDa) and N-glycosylated mature (∼110 kDa) forms of NEP was detected [5]. Addition of okadaic acid (OA) during labeling markedly increased the phosphate incorporation into NEP, indicating that phosphoprotein phosphatases (PP) 1 and/or 2 are involved in its dephosphorylation (Fig. 1B). In order to identify the phosphorylation sites of NEP we first analyzed its cytoplasmic domain by the NetPhos 2.0 server (Fig. 1A). cDNA constructs with mutations of the potential phosphorylation sites (Fig. 1A) within the cytoplasmic domain of NEP were generated and expressed in HEK293 cells. ^32^P-labeling revealed that substitution of serine residues 6 to alanine (S6A) strongly decreased phosphate incorporation. As compared to NEP wt, the phosphorylation of the Thr15Ala mutant appeared to be increased in independent experiments and might be due to effects of the mutation on substrate recognition (Fig. 1C). Together, these data indicate that serine 6 within the N-terminus is the main phosphorylation site of NEP.

Ser6 is localized within a consensus sequence for protein kinase CK2 (S-X-X-E/D) [43]. To test whether NEP is phosphorylated by CK2 *in vivo*, cells were labeled with ^32^P-orthophosphate in the presence or absence of the selective CK2 inhibitor DMAT. Cell treatment with DMAT markedly decreased the phosphorylation of NEP in cultured cells (Fig. 1D). Next we tested the phosphorylation of NEP by CK2 *in vitro*. CK2 efficiently phosphorylated the GST-NEP fusion protein, but not GST alone. Importantly, CK2-mediated phosphorylation of GST-NEP was strongly decreased in a Ser6Ala variant (Fig. 1E), demonstrating that CK2 predominantly phosphorylates serine 6 within the cytoplasmic domain of NEP.

**Figure 2:**
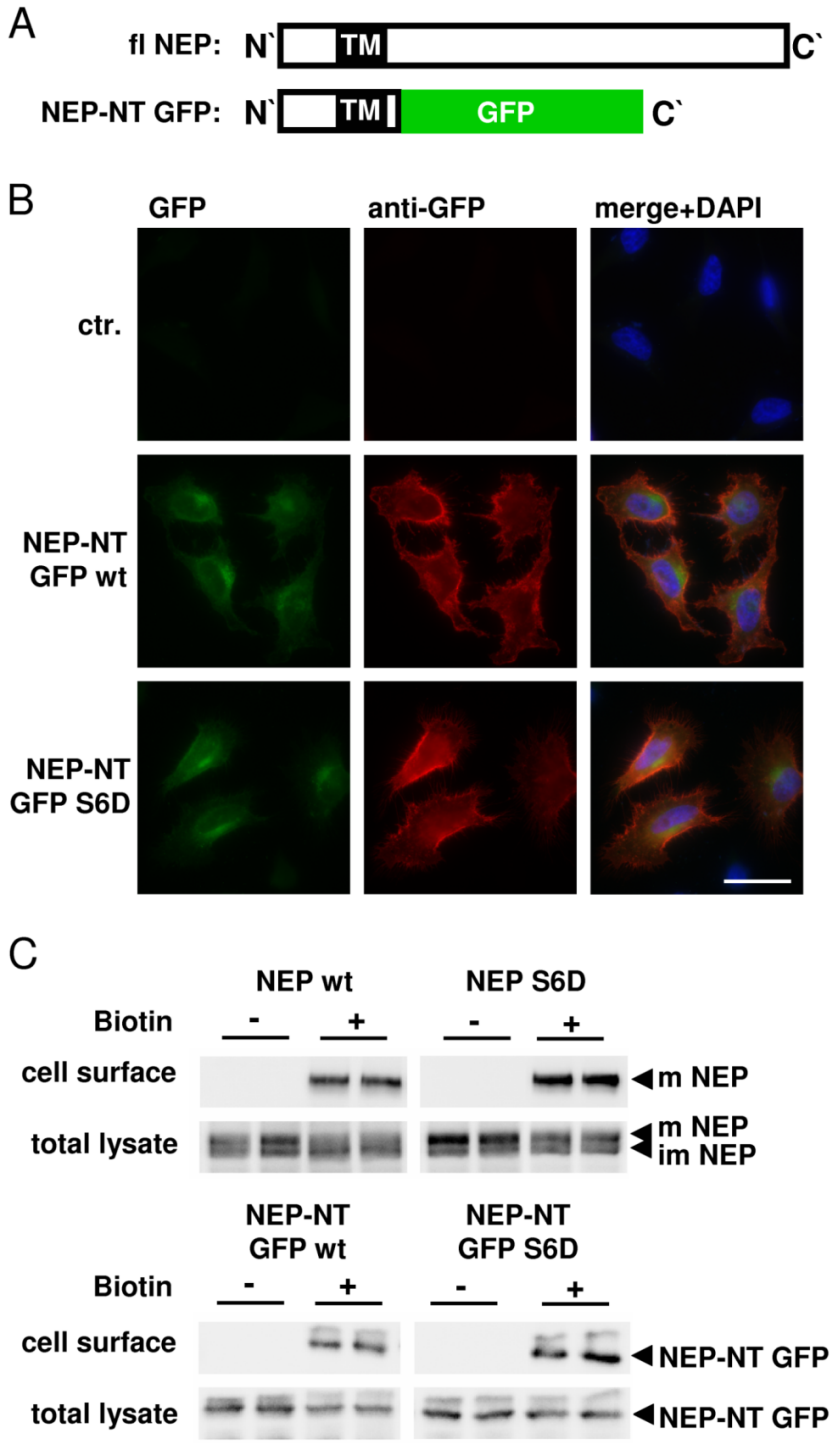
Localization of full-length NEP and GFP-tagged chimeric proteins at the cell surface. *A*, Schematic representation of full-length (fl) NEP and the chimeric NEP N-terminal GFP variant (NEP-NT GFP). *B*, Non-permeabilized HeLa cells expressing NEP-NT GFP wt or NEP-NT GFP Ser6Asp (S6D) were stained with mouse monoclonal antibody against GFP. The primary antibody was detected by Alexa 594-conjugated anti mouse secondary antibody. Images are representative for the typical distribution of NEP-GFP variants in several independent experiments. Note that NEP-NT GFP wt and the S6D Mutant are transported to the plasma membrane. *C*, HEK293 cells transiently expressing full-length NEP wt or NEP S6D and HEK293 cells stably expressing NEP-NT GFP wt or NEP-NT GFP S6D were cell surface biotin labeled. Biotinylated proteins were subsequently isolated with SA-agarose. All NEP variants are transported to the cell surface. Note the selective biotin labeling of the mature forms of full-length NEP. m = mature; im = immature; scale bar = 20 µm; ctr. = untransfected cells.

Because cell clones that stably express full-length catalytically active NEP could not be established (not shown), we generated NEP variants with substitutions of the extracellular catalytic domain by GFP (NEP-NT GFP; Fig. 2A). These constructs allowed the selection of cell clones that stably express NEP variants. In addition, the substitution of the extracellular domain of NEP by GFP allows to specifically assess functions of NEP that are independent of its proteolytic activity. To assess potential effects of the substitution of the ectodomain by GFP on the subcellular localization of NEP, we compared the distribution of NEP-NT GFP and full-length NEP by fluorescence microscopy. Both variants showed very similar distribution and localized to the endoplasmic reticulum and Golgi compartments (Fig. S1). These data demonstrate that substitution of the NEP ectodomain with GFP did not affect its subcellular trafficking and localization.

In order to mimic phosphorylated NEP we also generated a (pseudophosphorylated) Ser6Asp variant of NEP. As indicated in Fig. 2B, GFP signals were observed in juxtanuclear structures indicative for Golgi compartments for both NEP-NT GFP wild type (wt) and NEP-NT GFP Ser6Asp. Both variants were also detected at the plasma membrane indicating transport to the cell surface (Fig. 2B). The localization of NEP-NT GFP variants as well as the mature form of the full length NEP variants at the plasma membrane was also evident upon specific labeling of cell surface proteins with biotin (Fig. 2C). Thus, these NEP-NT GFP constructs proved suitable to study the role of the NEP N-terminus and its phosphorylation in cellular models.

**Figure 3:**
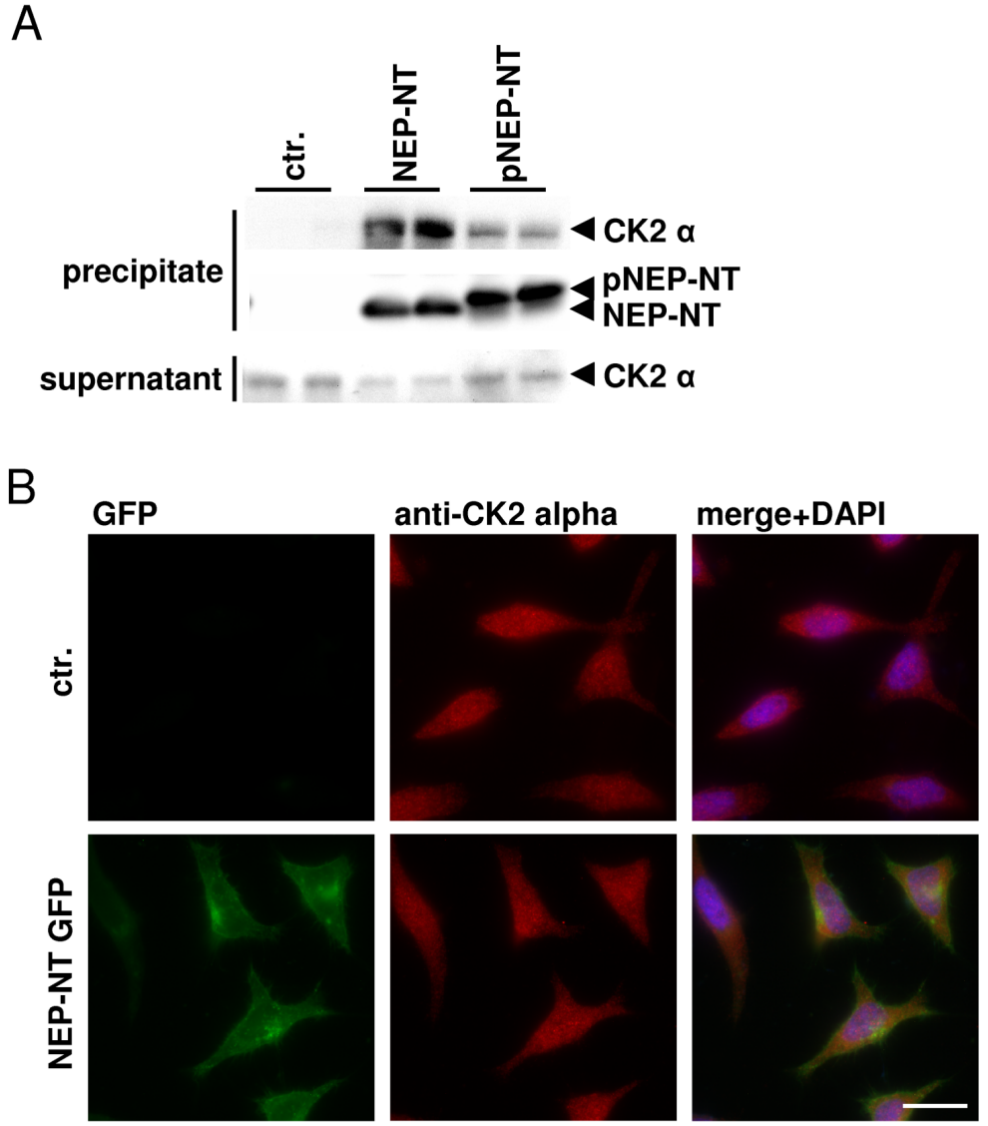
CK2 directly interacts with the N-terminus of NEP *in vitro.* *A*, Biotin-labeled peptides that represent the N-terminal domain of non-phosphorylated (NEP-NT) or phosphorylated (pNEP-NT) NEP were coupled to SA-agarose beads and incubated with purified recombinant CK2. Precipitated proteins were detected by western immunoblotting. *B*, HeLa cells transiently expressing NEP-NT GFP were stained with mouse monoclonal antibody against CK2 alpha. The primary antibody was detected by Alexa 594-conjugated anti mouse secondary antibody. Scale bar = 20 µm; ctr. (*A*) = no peptide; ctr. (*B*) = untransfected cells.

We next analyzed the interaction of CK2 and NEP by pull-down experiments. As demonstrated in Fig. 3A, recombinant CK2 efficiently co-precipitated with synthetic peptides that represent the cytosolic N-terminal domain of NEP (NEP-NT). Notably, this interaction was strongly decreased with a synthetic peptide containing Ser6 in a phosphorylated state (pNEP-NT; Fig. 3A). These data suggested that binding of CK2 decreased after phosphorylation of NEP. The subcellular localization of NEP and CK2 was then investigated by fluorescence microscopy. Consistent with previous data [44], CK2 showed broad distribution in the cytoplasm and also localization in the nucleus (Fig. 3A). NEP-NT GFP was mainly localized in juxtanuclear compartments and at the cell surface. However, there was no significant alteration in the localization of CK2 upon expression of NEP-NT GFP. These combined data indicate that CK2 transiently interacts with the N-terminus of NEP to mediate its phosphorylation.

### Phosphorylation of NEP selectively regulates the interaction with PTEN

**Figure 4:**
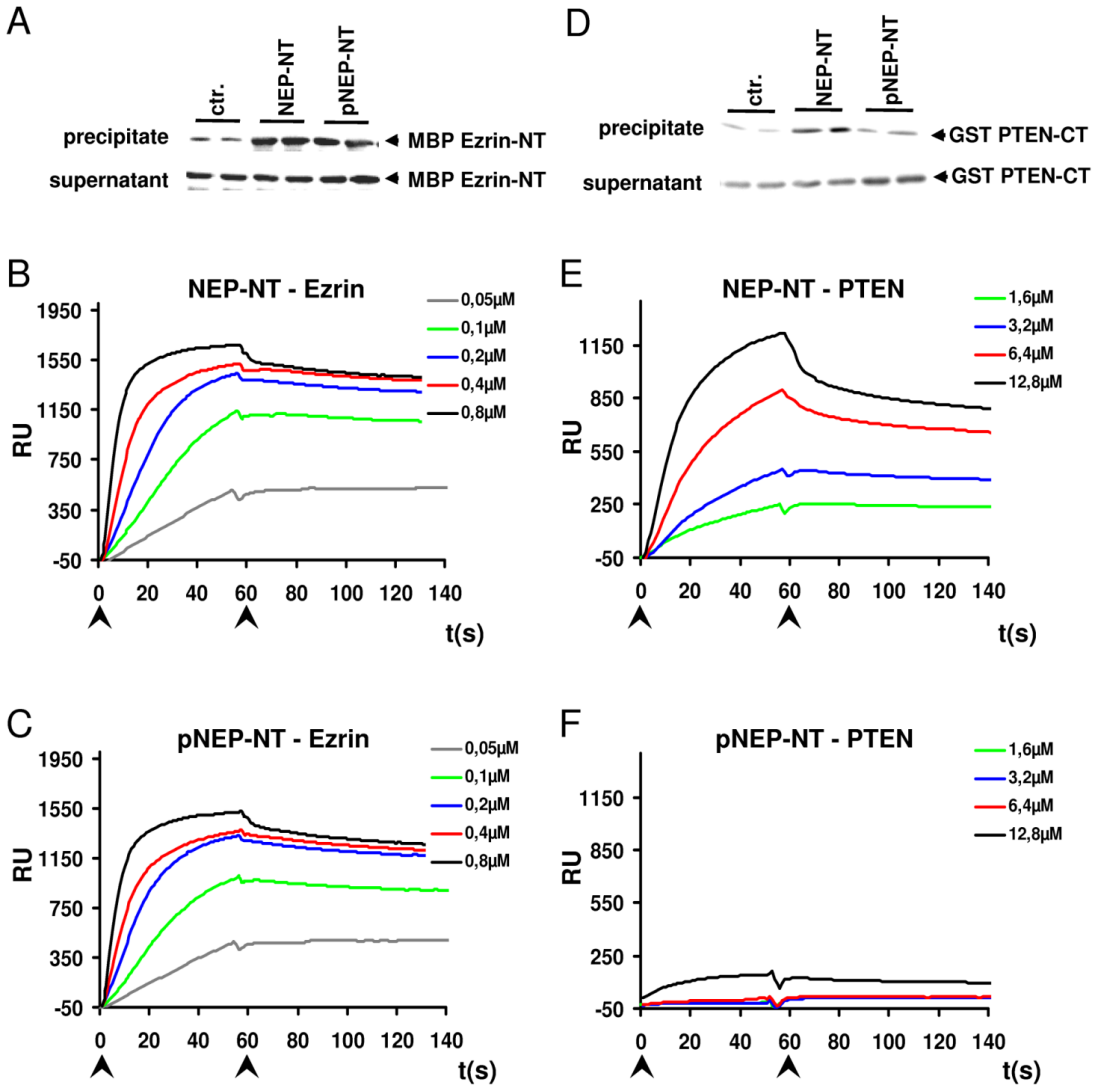
Phosphorylation of NEP selectively inhibits the interaction with PTEN. *A*, Biotin-labeled peptides that represent the N-terminal domain of non-phosphorylated (NEP-NT) or phosphorylated (pNEP-NT) NEP were coupled to SA-agarose beads and incubated with MBP Ezrin-NT (*A*) or GST PTEN-CT (*D*). SA-agarose precipitated proteins were detected by western immunoblotting. *B*, *C*, Sensograms of the interactions of MBP Ezrin-NT (concentrations ranging from 0.05 µM – 0.8 µM) with non-phosphorylated (NEP-NT; *B*) or phosphorylated (pNEP-NT; *C*) cytoplasmic domains of NEP. *E*, *F*, Sensograms of the interactions of GST PTEN-CT (concentrations ranging from 1.6 µM–12.8 µM) with non-phosphorylated (NEP-NT; *E*) or phosphorylated (pNEP-NT; *F*) cytoplasmic domains of NEP. Note that phosphorylation of NEP strongly inhibits the interaction with PTEN, while that with Ezrin is not affected. Arrows indicate start and end of injection, respectively; RU = response units; ctr. = no peptide.

The cytosolic domain of NEP interacts with the scaffolding proteins Ezrin/Radixin/Moesin (ERM) [7], [11] as well as the tumor suppressor PTEN [10]. To assess whether NEP phosphorylation affects these interactions, we first performed pull down assays with peptides representing the N-terminus of NEP in a non-phosphorylated and phosphorylated.state. Ezrin co-precipitated with both the phosphorylated and non-phosphorylated variants of NEP-NT (Fig. 4A). Surface plasmon resonance (SPR) spectroscopy revealed that Ezrin binds with very high affinity to the non-phosphorylated NEP-NT (K_D_∼5.8 nM). However, no significant difference in the binding of Ezrin to phosphorylated NEP-NT was observed (K_D_∼5.9 nM; Fig. 4B, C), indicating that the binding of Ezrin is independent of the phosphorylation state of NEP at serine 6.

Consistent with previous data [10], the NEP-NT also bound to the PTEN C-terminus in pull-down assays. Interestingly, this interaction was strongly decreased with the NEP-NT phosphorylated at Ser6 (Fig. 4D). SPR measurements fully supported these findings. While PTEN efficiently bound the non-phosphorylated NEP-NT (K_D_∼0,71 µM), the binding to the phosphorylated NEP-NT was strongly decreased (K_D_ could not be calculated; Fig. 4E, F). Together, these data demonstrate that the phosphorylation of NEP negatively regulates its interaction with PTEN.

**Figure 5:**
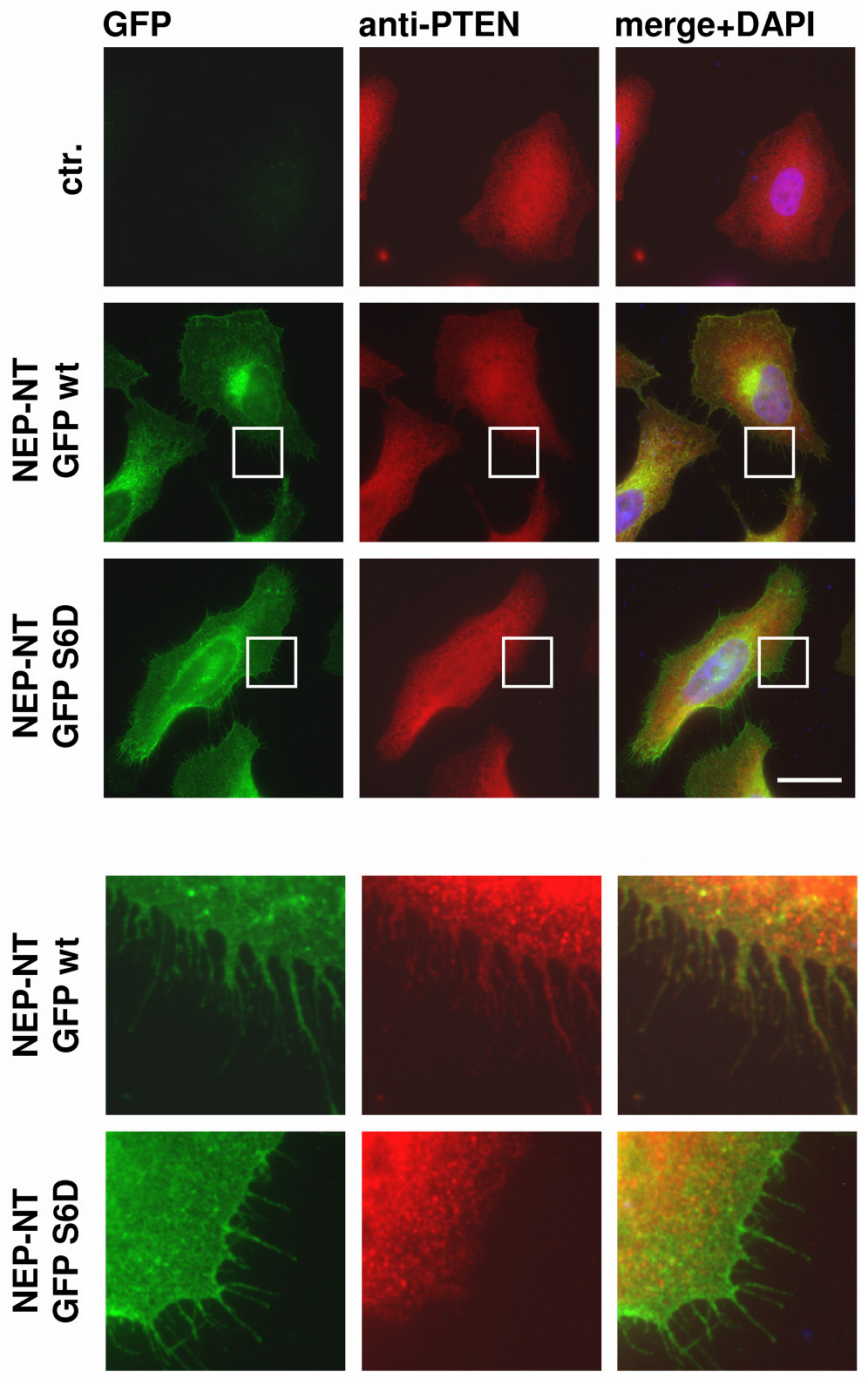
Decreased interaction of pseudophsphorylated NEP with PTEN at the cell surface. HeLa cells transiently expressing NEP-NT GFP wt or NEP-NT GFP S6D were stained with rabbit polyclonal antibody against PTEN and Alexa 594-conjugated anti rabbit secondary antibody. Note the selective co-localization of NEP-NT GFP wt and PTEN at the cell surface. Images are representative for the typical distribution of NEP-NT GFP variants and PTEN in several independent experiments. The boxed areas are enlarged in the lower panels. Scale bar = 20 µm; ctr. = untransfected cells.

To further investigate the effect of phosphorylation on the interaction of NEP and PTEN in cultured cells, we next analyzed the co-localization of endogenous PTEN with NEP-NT GFP wt and pseudophosphorylated NEP-NT GFP Ser6Asp variants. In non-transfected cells, PTEN revealed a broad distribution in the cytosol and the nucleus (Fig. 5, upper panels). In cells expressing NEP-NT GFP wt, PTEN was found to co-localize with NEP in juxtanuclear compartments. More importantly, there was also significant co-localization of NEP-NT GFP wt and PTEN at the plasma membrane (Fig. 5, middle panels and enlarged area). In contrast, very little if any co-localization of PTEN with the NEP-NT GFP Ser6Asp variant was observed at the plasma membrane (Fig. 5, lower panels and enlarged area). These data are consistent with our biochemical experiments and demonstrate that phosphorylation of NEP decreases the interaction with PTEN and its localization at the plasma membrane.

### Phosphorylation of NEP uncouples PTEN dependent inhibition of Akt

Recruitment of PTEN to the plasma membrane leads to increased dephosphorylation of PIP_3_ and attenuates the activation of Akt. To investigate whether the phosphorylation-dependent interaction of NEP and PTEN could affect the activation of Akt we used HEK293 cells stably expressing NEP-NT GFP wt and Ser6Asp variants (see also Fig. 2A).

**Figure 6:**
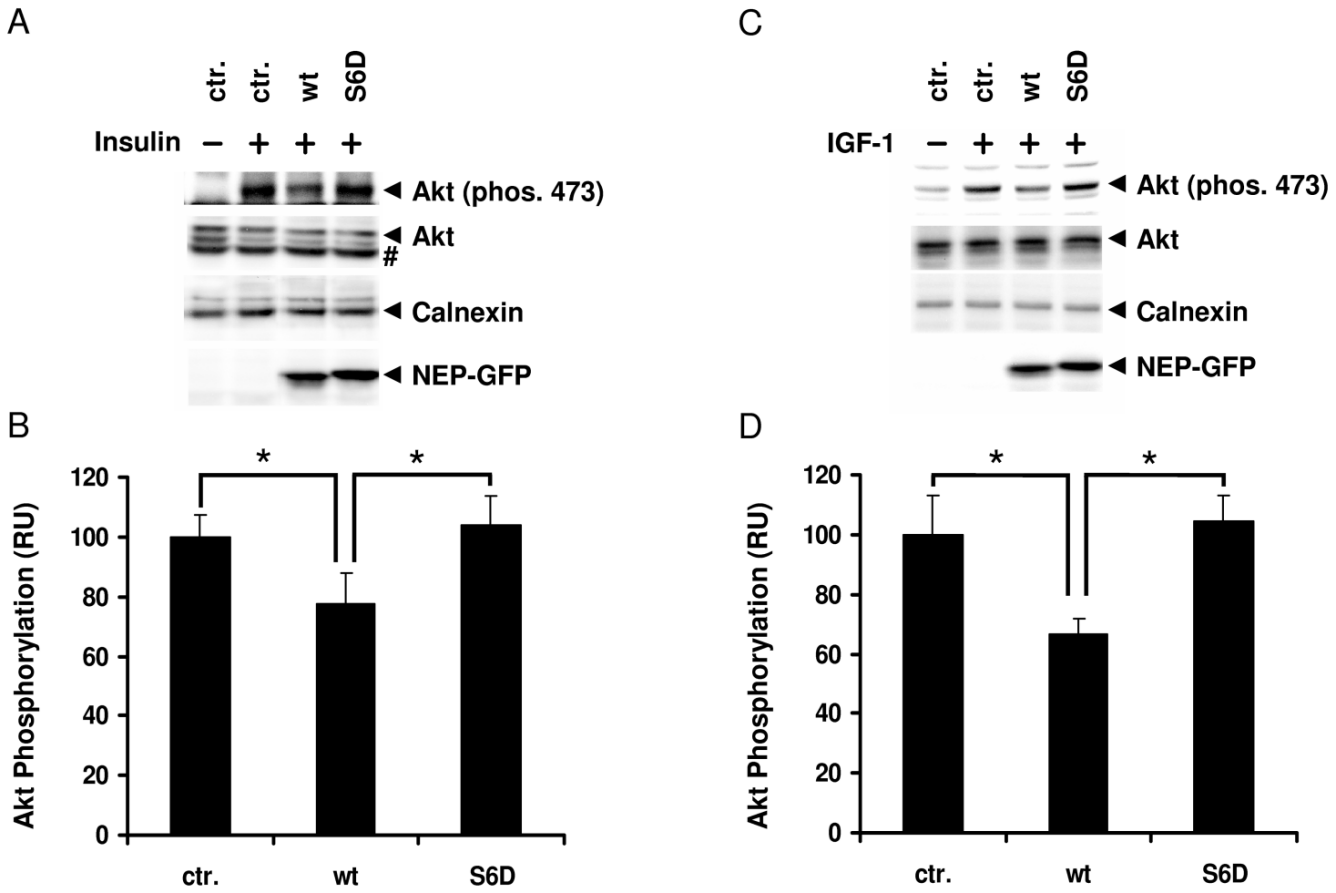
Selective inhibition of Akt activation by non-phosphorylated NEP upon stimulation with insulin or IGF-1. *A*–*D*, HEK293 cells stably expressing NEP-NT GFP wt or NEP-NT GFP S6D cells were incubated for 16 hrs in serum free medium and then treated with 100 nM insulin (*A*, *B*) or 1 ng/ml IGF-1 (*C*, *D*) for 20 min. Non-transfected cells served as controls. Cells were lysed and proteins were detected by western immunoblotting. Quantification of the Akt phosphorylation (serine 473) upon stimulation with insulin (*B*) or IGF-1 (*D*) was done by ECL imaging. Calnexin signals were used as loading control. Values represent means ± S.D. (n = 3); * *p*-value<0.05. # = unspecific band; ctr. = untransfected cells.

Cells were starved over night in media without serum and then treated with insulin to activate PI3K via insulin receptors. Incubation with insulin led to a time-dependent increase in the phosphorylation of Akt indicating rapid activation of PI3K. After 20 min, the phosphorylation of Akt reached a maximum in untrasfected cells (data not shown). Expression of NEP-NT GFP wt significantly attenuated the phosphorylation of Akt by 23%, indicating recruitment of PTEN to the cytoplasmic domain of NEP and increased dephosphorylation of PIP_3_ (Fig. 6A, B). Interestingly, the pseudophosphorylated NEP-NT GFP Ser6Asp variant had no inhibitory effect on insulin-dependent activation of Akt (Fig. 6A, B). Very similar data were also obtained when IGF-1 was used to stimulate the phosphorylation of Akt (Fig. 6C, D).

## Discussion

Our data demonstrate that CK2 phosphorylates NEP and thereby regulates the recruitment and activity of PTEN at the plasma membrane. Previous data also indicated that NEP can be phosphorylated by CK2 *in vitro* [36], but the phosphorylation site and functional relevance was unknown. We identified Ser6 as a major phosphorylation site within the cytoplasmic domain of NEP *in vitro* and in cultured cells.

Ser6 and the surrounding amino acid sequence are highly conserved in mammalian variants of NEP and constitute a canonical recognition motif for CK2 (see Fig. 1A). In addition, specific inhibition of CK2 by DMAT inhibits NEP phosphorylation (Fig. 1D).

CK2 is ubiquitously expressed and, in contrast to most other protein kinases, a constitutively active enzyme. Its regulation is not well understood and appears to be independent of known second messengers [45]. Consistent with the large number of protein substrates, CK2 serves pleiotropic functions in cellular signaling and metabolism [46], [47]. Accumulating evidence suggests that CK2 promotes cell proliferation and growth [48]. While the underlying mechanisms are not entirely clear, the phosphorylation of important signaling molecules, including p53, IκB, and kinases of the MAP/ERK family might be involved in the CK2-dependent regulation of the cell cycle [49], [50], [51], [52]. In addition, CK2 has also been implicated in insulin signaling. Levels of cytosolic CK2 were increased upon insulin injection [53], and found to be reduced in insulin resistant rats [54].

Our data demonstrate that the CK2-dependent phosphorylation of NEP could affect insulin/IGF-1 dependent intracellular signaling. Importantly, the phosphorylation of the cytosolic domain of NEP strongly decreased the interaction with the tumor suppressor PTEN. As shown previously, PTEN binds to a stretch of basic amino acids (aa 19–21) close to the transmembrane of NEP by electrostatic interaction [10]. While the identified phosphorylation site is localized outside of the binding domain of NEP for PTEN, CK2 dependent phosphorylation might induce a structural change of the NEP N-terminus. This notion is supported by the finding that synthetic peptides representing the N-terminal domain in a phosphorylated and non-phosphorylated state had distinct migration behaviour in SDS gels (Fig. 3A). In addition, the substitution of Ser6 by an aspartate residue also changed the migration of GFP-tagged NEP variants (Fig. 2C, 6A, 6C). Interestingly, amino acids 12–15 that could form a hairpin structure in the NEP N-terminus are localized between the identified phosphorylation site and the binding domain for PTEN [11]. Thus, phosphorylation of Ser6 might lead to the electrostatic interaction with a stretch of basic amino acids (aa 19–21) and thereby inhibit the interaction with PTEN. In contrast, the interaction of Ezrin with NEP was independent of its phosphorylation state. While Ezrin was initially considered to bind to the same region as PTEN, recent data indicate that Ezrin interacts with amino acids Met8, Ile10, Thr11, Ile13 and Asn14 [7], [11]. Thus, phosphorylation of Ser6 might not affect the conformation of this domain. It remains to be determined whether PTEN and Ezrin can bind simultaneously to NEP.

The physiological and pathophysiological relevance of PTEN is documented by its association with tumorgenesis. Beside p53, PTEN is the most important tumor suppressor and found to be inactivated in many human cancers [15], [17]. Because PTEN dephosphorylates PIP_3_, decreased activity results in elevated PIP_3_ levels thereby overactivating Akt signaling. An involvement of NEP in Akt signaling has emerged only recently [10]. The cytoplasmic domain of NEP could directly associate with PTEN at the plasma membrane and regulate its local activity. Consistent with previous results, this effect is independent of the catalytic activity of NEP, suggesting that the extracellular and intracellular domains of NEP could serve independent functions [10]. The NEP N-terminus also affects tumor formation by competing with the hyaluronan receptor CD44 for Ezrin/Radixin/Moesin (ERM) binding thereby reducing the invasive capacity of cancer cells [7].

In addition to NEP, PTEN is also phosphorylated by CK2. This phosphorylation leads to an increased stability of PTEN, but decreases its catalytic activity [29], [55]. Recent data demonstrated that rather non-phosphorylated PTEN interacts with NEP *in vivo* and that phosphorylation of PTEN decreases its binding to phospholipids in cellular membranes [10], [56]. Thus, CK2 could negatively regulate the activity of PTEN at the plasma membrane by phosphorylation of both PTEN and NEP allowing an effective and fine-tuned control of PTEN dependent signaling.

**Figure 7:**
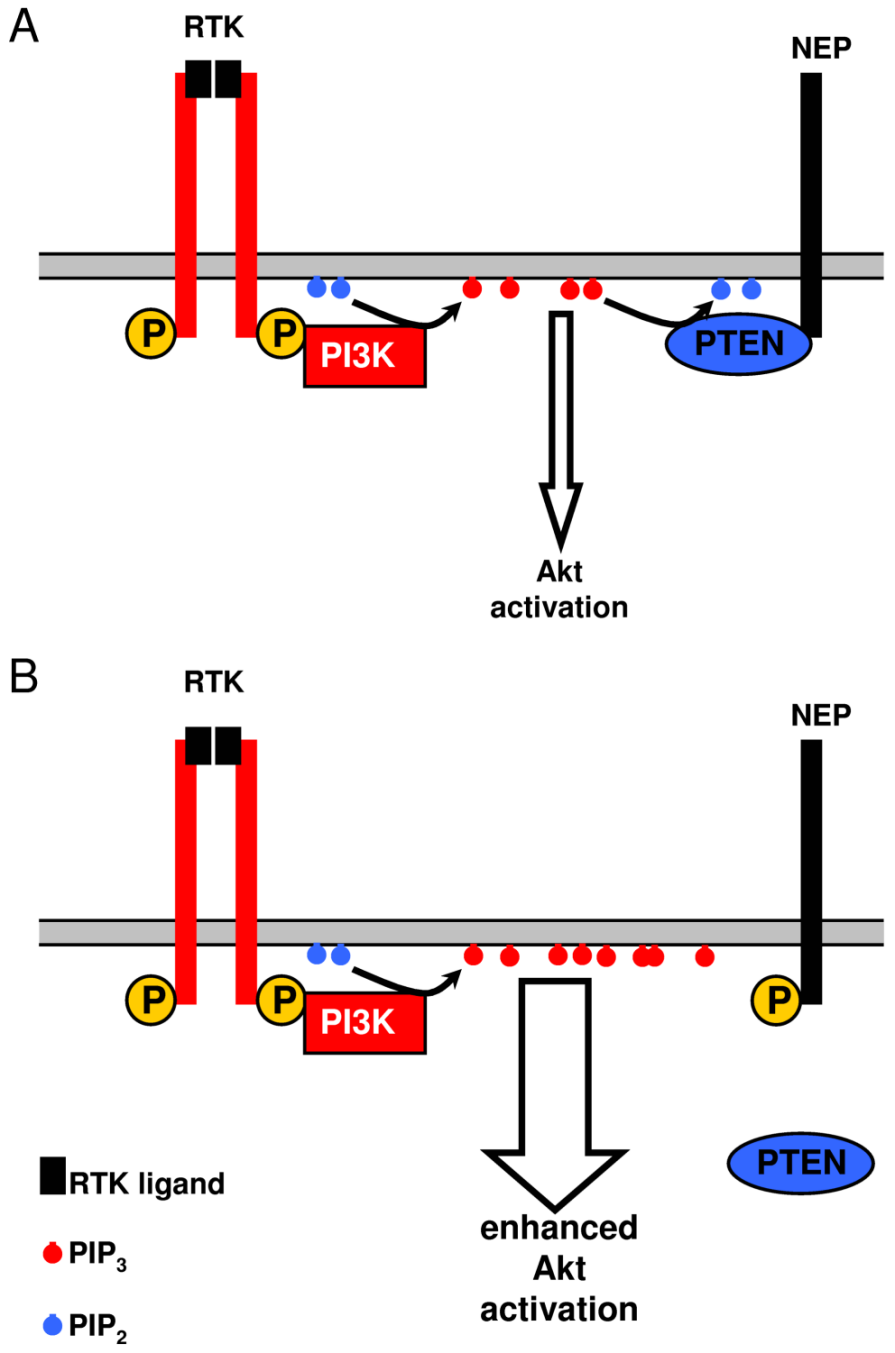
Model for the role of NEP phosphorylation in Akt signalling. PI3K is recruited to RTKs upon stimulation with specific ligands and phosphorylates PIP_2_ to PIP_3_. *A*, Non-phosphorylated NEP interacts with PTEN that antagonizes PI3K by the dephosphorylation of PIP_3_ to PIP_2_ resulting in the decreased Akt activation. *B*, The phosphorylation of NEP inhibits recruitment of PTEN to the plasma membrane and thereby stabilizes the pool of PIP_3_. Thus phosphorylation of NEP could increase or prolong the activation of Akt.

In cellular models, we demonstrate that phosphorylation of NEP attenuates down-regulation of RTK signaling to Akt. While the expression of a NEP wt variant decreased activation of Akt by insulin and IGF-1, a pseudophosphorylated (Ser6Asp) mutant had no significant effect. Thus, increased phosphorylation of NEP by CK2 could inhibit the dephosphorylation of PIP_3_ at the plasma membrane and thereby lead to prolonged activation of Akt by insulin, IGF-1 and possibly other RTK ligands (Fig. 7). Thus, overactivation of Akt by increased phosphorylation of NEP could enhance RTK-dependent signaling, and thereby survival or proliferation. It will therefore be interesting to further investigate the role of NEP phosphorylation in these processes *in vivo*.

## Supporting Information

Figure S1 Similar distribution of full-length NEP and NEP-NT GFP. HeLa cells were transiently transfected with cDNAs encoding full-length myc-tagged NEP or NEP-NT GFP. Myc-tagged NEP was detected by staining with mouse monoclonal antibody 9E10 and Alexa 488-conjugated anti mouse secondary antibody. The localization of NEP-NT GFP was analyzed by direct fluorescence microscopy. Cells were co-stained with polyclonal antibodies against TGN46 (A) or calnexin (B) and Alexa 594-conjugated anti rabbit secondary antibody to localize the trans-Golgi network and endoplasmic reticulum, respectively. Both full-length myc-tagged NEP and NEP-NT GFP showed very similar distribution in the trans-Golgi network (A) and the endoplasmic reticulum (B). Images are representative for the typical distribution of full-length myc-tagged NEP and NEP-NT GFP in independent experiments. Scale bar  =  20 μm.
(4.47 MB TIF)
