# Supplementary figures and images for: Casein Kinase 2 Dependent Phosphorylation of Neprilysin Regulates Receptor Tyrosine Kinase Signaling to Akt
Source: PLoS One. 2010 Oct 1;5(10):e13134. doi: 10.1371/journal.pone.0013134 (PMC2948513; doi:10.1371/journal.pone.0013134)

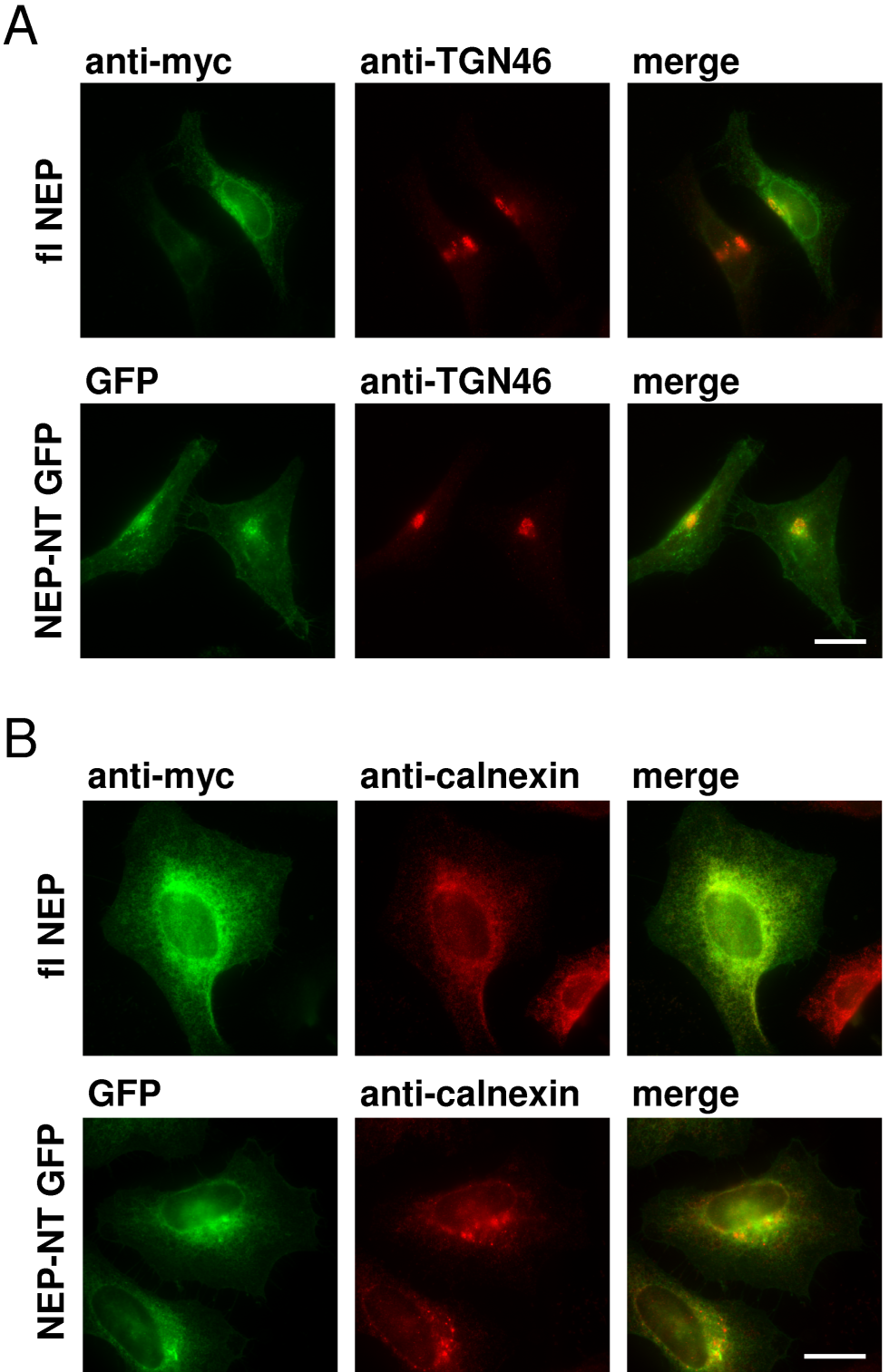

Supplement: Figure S1 — Similar distribution of full-length NEP and NEP-NT GFP. HeLa cells were transiently transfected with cDNAs encoding full-length myc-tagged NEP or NEP-NT GFP. Myc-tagged NEP was detected by staining with mouse monoclonal antibody 9E10 and Alexa 488-conjugated anti mouse secondary antibody. The localization of NEP-NT GFP was analyzed by direct fluorescence microscopy. Cells were co-stained with polyclonal antibodies against TGN46 (A) or calnexin (B) and Alexa 594-conjugated anti rabbit secondary antibody to localize the trans-Golgi network and endoplasmic reticulum, respectively. Both full-length myc-tagged NEP and NEP-NT GFP showed very similar distribution in the trans-Golgi network (A) and the endoplasmic reticulum (B). Images are representative for the typical distribution of full-length myc-tagged NEP and NEP-NT GFP in independent experiments. Scale bar = 20 μm. (4.47 MB TIF) [file pone.0013134.s001.tif]
